# POLQ knockdown inhibits proliferation, migration, and invasion by inducing cell cycle arrest in colorectal cancer

**DOI:** 10.1007/s12672-024-01496-x

**Published:** 2024-11-09

**Authors:** Qing Yao, Shuyang Gao, Qiannan Sun, Liuhua Wang, Jun Ren, Daorong Wang

**Affiliations:** 1 The Yangzhou School of Clinical Medicine of Dalian Medical University, Yangzhou, 225001 China; 2 Northern Jiangsu People’s Hospital, Yangzhou, 225001 China; 3 General Surgery Institute of Yangzhou, Yangzhou University, Yangzhou, 225001 China; 4 Yangzhou, Key Laboratory of Basic and Clinical Transformation of Digestive and Metabolic, Yangzhou, 225001 China; 5 Medical Research Center of Northern Jiangsu People’s Hospital, Yangzhou, 225001 China

**Keywords:** Polymerase θ, Colorectal cancer, Cell cycle signaling pathway, DNA replication

## Abstract

**Background:**

Polymerase θ (POLQ) is an error-prone translesion synthesis polymerase that participates in the repair of DNA double-strand breaks. Previous studies have reported that the level of POLQ expression is distinctly upregulated in colorectal cancer (CRC), but little attention has been given to its function and regulation of CRC progression. This study aimed to explore the specific function of POLQ in CRC.

**Methods:**

Quantitative real-time PCR and western blotting analysis were used to assess the transcription and translation levels of POLQ. Then, POLQ was stably silenced using small interfering RNA in SW480 and HCT116 cells. Afterwards, the function of POLQ in CRC cells was proven via Cell Counting Kit‑8, scratch wound healing, colony formation, and Boyden chamber assays. Furthermore, we investigated the effects of POLQ on the cell cycle signaling pathway that obtained from biological pathway enrichment analysis and further verified by activating the signaling pathway.

**Results:**

The results showed that POLQ was highly expressed in CRC tissues and cells and was associated with poor clinical outcomes of patients. Knockdown of POLQ significantly reduced the proliferation, migration and invasion of CRC cells. Additionally, POLQ knockdown markedly decreased the expression levels of MMP2 and MMP9, and blocked cell cycle progression by inhibiting the expression of G1/M and S/M phases proteins.

**Conclusions:**

POLQ knockdown restrained the progression of CRC by blocking the cell cycle signaling pathway.

## Introduction

Colorectal cancer (CRC), one of the most common cancers in the digestive system, ranks second in incidence and second in mortality according to the Cancer Statistics Report in China in 2020 [1]. Previous studies have demonstrated that the 5-year survival rate for patients with stage I colorectal cancer is 90%, compared with only 10% for those with stage IV colorectal cancer [2]. Presently, surgery and chemoradiotherapy are still commonly used to treat CRC. However, approximately 550,000 people die from CRC worldwide each year, which is closely connected with its characteristics of hidden onset, rapid progression, and lack of effective diagnosis and therapy [3]. Therefore, in-depth understanding of the pathogenesis of colorectal cancer and discovering effective targeted therapy are urgently needed to prolong the survival of CRC patients.

In mammalian cells, DNA polymerase θ is encoded by the gene POLQ, whose structure contains an N-terminal helicase-like region linked to the C-terminal DNA polymerase via a central region [4, 5]. POLQ is closely related to cell cycle control and DNA replication, and participate in the repair of DNA double-strand breaks (DSBs) [5]. Recent studies have shown that POLQ is significantly up-regulated in hepatocellular carcinoma, lung cancer, breast cancer, ovarian cancer, prostate cancer and other malignant tumors, and is closely related to poor clinical outcomes of patients [6–10]. However, there are few studies on the expression and prognosis of POLQ in CRC patients.

In this study, we mainly analyzed the expression level of POLQ in CRC tissues and cells and explored the effects of POLQ on the proliferation, migration, and invasion of CRC cells. Further research was conducted on how POLQ affected cell cycle signaling pathway to promote the deterioration of CRC. Therefore, we aimed to provide some fundamental theories for diagnosing and treating CRC.

## Materials and methods

### Bioinformatics analysis

The differential gene expression levels of POLQ in different types of tumors and normal tissues were obtained from TCGA. The significance of expression differences was calculated by the Wilcoxon test (*p < 0.05, **p < 0.01, ***p < 0.001). To provide clinicians with a quantitative method of predicting the prognosis of patients with colon adenocarcinoma (COAD), we constructed a nomogram based on traditional clinical characteristics to predict survival rates. Univariate Cox analyses were first performed. Variables with p < 0.05 were subsequently included in multivariate Cox regression. Finally, a nomogram was constructed using the 'rms' package based on the results of the multivariate Cox regression analysis.

R statistical software was applied to analyze co-expressed genes associated with POLQ. To explore the biological functions of differentially expressed genes in POLQ co-expressed genes, we performed functional enrichment analysis using the “Cluster-profiler” package, including GO (Gene Ontology) and KEGG (Kyoto Encyclopedia of Genes and Genomes) analysis. The screening criteria were P < 0.05 and FDR < 0.05. CIBERSORT was used to assess the scores of 22 immune cells in each COAD patient. The Wilcoxon signed-rank test was used to evaluate the difference in immune cell infiltration between the high and low POLQ expression groups, with a significance threshold of P < 0.05. To investigate the potential impact of POLQ on the response to immunotherapy, we analyzed the differences in the expression of key immune checkpoints between the high and low levels of POLQ expression groups. The IC50 values of drugs for COAD and the differences in drug sensitivity between patients with high and low levels of POLQ expression were calculated using the 'prophetic' package in R statistical software.

### Patients and specimens

Forty-eight colorectal cancer tissues and paired para-carcinoma tissues were collected from patients with CRC at Northern Jiangsu People's Hospital. Paired para-carcinoma tissues were obtained from at least 5 cm away from the tumor. Tissues for IHC were stored in formalin immediately after being isolated. Before immunohistochemistry, paraffin sections or tissue microarrays were made. The tissues used for WB were stored in liquid nitrogen immediately after being isolated and then stored in a refrigerator at -80° C. The age of the patients ranged from 39 to 91, and there were 28 male patients and 20 female patients. Patients were diagnosed with CRC between July 2015 and May 2017 and had not received preoperative radiotherapy or chemotherapy. All tissues were adjudicated by at least two qualified pathologists. The American Joint Committee on Cancer TNM staging system (AJCC-8 TNM) was used to identify the stage of CRC. Clinical data of the patients were obtained from hospital databases and clinical records. Informed consent was obtained from the patients or their families for all clinical and follow-up data. This study was approved by the Ethics Committee of Northern Jiangsu People's Hospital, and informed consent was obtained from all participants.

### Cell culture

Normal intestinal epithelial cell lines (NCM460) and CRC cell lines (SW480, HCT116, SW620) were purchased from the Cell Bank of Culture Collection of Chinese Academy of Sciences (Shanghai Institute of Cell Biology, Chinese Academy of Science). The NCM460 and HCT116 cell lines were cultured in McCoy's 5A medium (Pricella, Procell, China). The SW480 and SW620 cell lines were cultured in RPMI-1640 medium (Gibco, Thermo Fisher Scientific, China) and DMEM/high glucose medium (Gibco; Thermo Fisher Scientific, China), respectively. The cultivation of all cell lines was supplemented with 10% fetal bovine serum (Gibco, Thermo Fisher Scientific, China) and 1% penicillin/streptomycin (Solarbio, Beijing, China) at 37 °C in a humidified incubator with a 5% CO2 atmosphere.

### Immunohistochemistry (IHC)

Immunohistochemical analysis was performed on CRC tissues and paired para-carcinoma tissues from 48 confirmed patients using a tissue microarray (TMA). The sections were placed in an oven at 60 °C for 2 h before deparaffinization with xylene and hydration with gradient concentrations of ethanol (100–60%). Subsequently, the sections were heated in sodium citrate antigen retrieval solution (Solarbio, Beijing, China) for 30 min, and then endogenous peroxidase activity was blocked with 3% hydrogen peroxide. Sections were incubated with POLQ polyclonal anti-rabbit antibody (1:50; Cat no. D263727; Sangon Biotech) overnight at 4 °C. The next day, the sections were incubated with biotin-labeled antibodies and horseradish peroxidase (HRP) for 30 min at 37 °C. Then, the sections were visualized with freshly configured DAB solution and counterstained with hematoxylin. Finally, the sections were dehydrated with gradient concentrations of alcohol and xylene and captured under an Olympus BX53 fluorescence microscope (Olympus Corporation). Staining intensity was scored as follows: 0 for colorless, 1 for yellow, 2 for brown, and 3 for dark brown. The score was based on the percentage of cells: 0 for 0–5%, 1 for 5–25%, 2 for 25–50%, 3 for 50–75%, and 4 for > 75%. The sum of staining intensity and cell percentage was used as the final score. Cases with a score < 5 were classified as having low POLQ expression, and those with a score greater than or equal to 5 were classified as having high POLQ expression.

### Cell transfection

1. CRC cells in good culture condition were digested, collected, counted and seeded in six-well plates at a rate of 2 × 105 cells/well. CRC cells were replaced with new medium (without serum and double antibody) and cultured in the cell incubator when the cells grew to about 70% of the area of the six-well plate. Then, the cells were cultured in a cell incubator and used for transfection when the medium temperature reached 37℃.
2. 5µL siRNA was dissolved in 45µL medium (without serum and double antibody), gently blown and mixed, and allowed to stand for 5 min at room temperature.
3. 5µL LipofectamineTM 2000 was dissolved in 45µL culture medium (without serum and double antibodies), gently blow and mix, and allowed to stand for 5 min at room temperature.
4. After the end of standing, drop the mixture in step 2 into the mixture in step 3 (a meter-drop of 5µL per time), gently blow and mix, and let it stand for 20 min at room temperature.
5. The six-well plate in step 1 was removed, and the mixture containing siRNA and LipofectamineTM 2000 in step 4 was uniformly dropped into the six-well plate (10µL of each metered-drop), labeled and cultured in a cell incubator.
6. After the cells were grown in the incubator for 6–8 days, they were replaced with new complete medium. Then, they were placed in incubators for growth for 48 h before being used for subsequent experiments.

The following target sequences were created for POLQ: shPOLQ forward, 5′-CCAUGUGCCCUGUUACAUUTT-3′ and reverse, 5′-AAUGUAACAGGGCACAUGGTT-3′. Besides, the cell cycle agonist DMT-dC(ac) phosphoramidite (MedChemExpress, Shanghai, China) was transfected into HCT116 cells at a concentration of 5 nM to activate the cell cycle signaling pathway. Dimethyl sulfoxide (DMSO) (Solarbio, Beijing, China) was applied in the experiments as a solvent for agonist at a concentration of 0.05%.

### Quantitative real-time PCR (qRT‒PCR)

Total RNA was extracted from cell lysates using Trizol reagent (Invitrogen; Thermo Fisher Scientific, China). The extracted RNA was reverse transcribed using a cDNA Synthesis kit and then used as a template for qRT‒qPCR with 2 × Universal SYBR Green Fast qPCR Mix (Cat no. rk21203, ABclonal, China) according to the manufacturer's instructions. The relative gene expression levels were calculated using the 2^−∆∆Ct^ method, with GAPDH as the reference gene. The primer sequences were as follows: GAPDH, forward, 5′-TGACATCAAGAAGGTGGTGAAGCA-3′ and reverse, 5′-GTGTCGCTGTTGAAGTCAGAGGAG-3′; and POLQ forward, 5′-GTGAAGACCCGTTTACCATAGA-3′ and reverse, 5′-AGATCCTGTGACAATATGCTCC-3′.

### Western blotting (WB) analysis

Proteins were obtained by lysis of the cells with RIPA lysis buffer (Cat no. R0020; Solarbio, China). Samples were mixed with 5X loading buffer solution and thenboiled for 7 min after determining the protein concentration using a BCA kit (Cat no. P0012; Beyotime Institute of Biotechnology). Then, equivalent extracted amounts of proteins (30 µg protein per lane) were separated by 10% SDS‒PAGE and electrotransferred to polyvinylidene fluoride (PVDF) membranes (Thermo Fisher Scientific, China). After being blocked with 5% skim milk for two hours at room temperature, the membranes were incubated with primary antibodies overnight at 4 °C. After being washed three times with TBST, the membranes were incubated with horseradish peroxidase–conjugated secondary antibody for two hours at room temperature. Finally, the membranes were detected for the signal of Enhanced Chemiluminescent Substrate (Cat no. P10300; New Cell & Molecular Biotech Co. Ltd). The antibodies used in the experiment were as follows: rabbit anti-POLQ (1:1000; cat no. PA5-115130, Sangon Biotech), mouse anti-GAPDH (1:10,000. cat no. HRP-60004, Proteintech), rabbit anti-CDK4 (1:1000. Cat no. 11026-1-AP, Proteintech), rabbit anti-CDK6 (1:1000. Cat no. 14052-1-AP, Proteintech), rabbit anti-cyclin D1 (1:1000. Cat no. A11022, ABclonal), rabbit anti-MCM2 (1:2000. Cat no. D120962, Sangon Biotech), rabbit anti-MCM7 (1:2000. Cat no. 220965, Sangon Biotech), rabbit anti-MMP2 (1:1000. Cat no. 10373-1-AP, Proteintech), rabbit anti-MMP9 (1:1000. Cat no. 27306-1-AP, Proteintech).

### Cell counting Kit‑8 (CCK‑8)

Cells were incubated in a 96-well plate at a density of 1 × 103 cells per well after siRNA transfection for 48 h. After incubation in the incubator for 24 h, 48 h, and 72 h, 110 µl of diluted CCK-8 solution (100 µL serum-free medium + 10 µl CCK-8 reagent) was added to each well. Then, the optical density (OD) values of each cell were measured at 450 nm after reaction for two hours at a temperature of 37 °C.

### Colony formation

Cell suspensions were made after 48 h of siRNA transfection. Six-well plates were plated with 600 cells per well and cultured in incubators for 14 days. The colonies were stained with 0.1% crystal violet for 30 min after being fixed with 4% paraformaldehyde for 15 min at room temperature. Finally, the colonies were photographed with a digital camera and counted via ImageJ software.

### Wound healing assay

Uniform wounds were generated in a monolayer of CRC cells in small Petri dishes using a 1000 µl pipette tip vertically. The exfoliated cells were washed twice with PBS. Cells were sequentially cultured with culture medium containing 3% FBS at 37 °C in an incubator with 5% CO2. Then, cells in small petri dishes were photographed at 0, 24, and 48 h with a light microscope. The area of cell migration was calculated using ImageJ software.

### Migration and invasion assays

After 48 h of transfection, cells were digested in small Petri dishes and suspended in serum-free medium at a density of 1 × 105 cells/ml. Two hundred microliters of cell suspension was added to 8 µm Boyden chambers (Corning) placed in 24-well plates. Then, 500 µl of medium containing 10% FBS was added to the lower chamber. For the invasion assay, the diluted Matrigel matrix gel (serum-free medium: Matrigel matrix gel, 9:1) was added to Boyden chambers and placed in the incubator for 24 h and used in the following experiment after it became solid. After 48 h of incubation at 37 °C, 4% paraformaldehyde was added to the chamber to fix the cells for 30 min. Fixed cells were stained with 0.1% crystal violet (100 ml methanol: 0.1 g crystal violet) for 15 min. Then, the chambers were washed three times with PBS after removing cells that had not passed through the chamber with cotton swabs. Finally, six fields of view for each chamber were selected randomly and photographed under a light microscope. Cells were counted using ImageJ software.

### Statistical analysis

SPSS Statistics 26 software was used to analyze the data. All experiments were repeated at least three times. Data are expressed as the mean ± standard deviation. The t test was used when the quantitative data of the two groups were in accordance with normal distribution and homogeneity of variance. Statistical analysis of POLQ expression and clinical data was performed using the chi-square test. The Kaplan‒Meier method and log-rank test were used to analyze the relationship between POLQ expression and the prognosis of patients. Cox proportional hazard regression was used to analyze the independent prognostic factors. P < 0.05 indicates that the difference is statistically significant (* p < 0.05, ** p < 0.01, *** p < 0.001).

## Results

### Characteristics of the POLQ gene in bioinformatics analysis

**Fig. 1:**
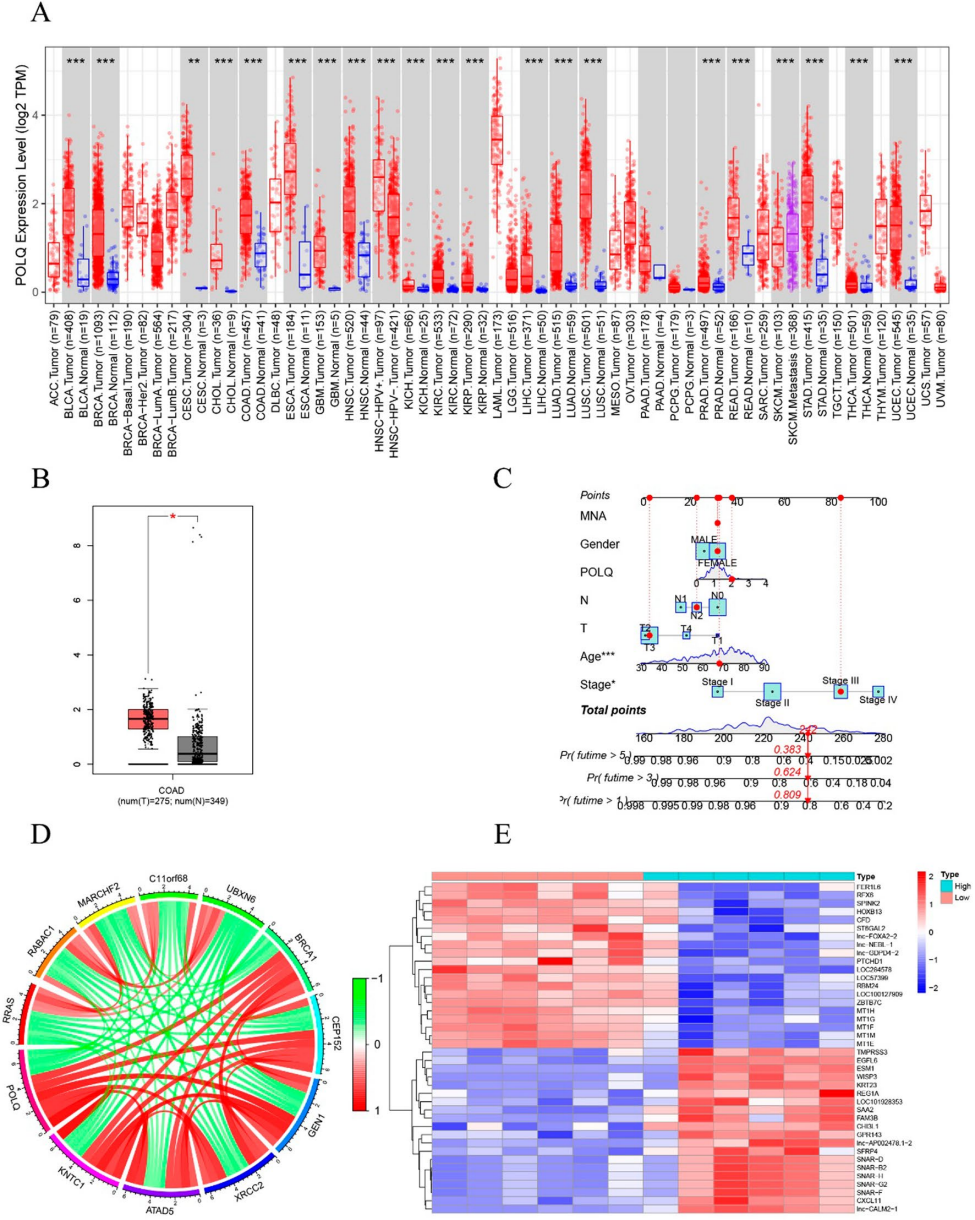
The expression of POLQ and its co-expressed genes in bioinformatics analysis. **A** Distribution of POLQ expression levels between tumor and normal tissues in various types of cancer. **B** Differential expression levels of POLQ in COAD. **C** A predictive nomogram for OS in COAD patients. **D** A total of 251 POLQ-associated co-expressed genes. **E** Heatmap showing the distribution of POLQ co-expressed genes. *P < 0.05, **P < 0.01, ***P < 0.001

We obtained the expression level of POLQ from a pan-cancer perspective through the TCGA database, which showed a statistical overexpression of POLQ in 21 types of malignancies (Fig. 1A). Meanwhile, we acquired the results of the differential expression of POLQ in COAD through the GEPIA database (Fig. 1B). A predictive nomogram for OS in COAD patients was constructed based on POLQ expression, gender, T stage, N stage, age, and stage. The results showed that age and stage were significantly associated with the prognosis of COAD (Fig. 1C). Then, we identified 251 POLQ-associated co-expressed genes through the construction of a gene co-expression network, which showed that a series of genes, including KNTC1, ATAD5, XRCC2, GEN1, CEP152 and BRCA1, were positively correlated with POLQ and other negatively related genes, including RRAS, RABAC1, MARCHF2 and C11orf68 (Fig. 1D). A heatmap shows the distribution of POLQ co-expressed genes in the high and low POLQ expression groups (Fig. 1E).

**Fig. 2:**
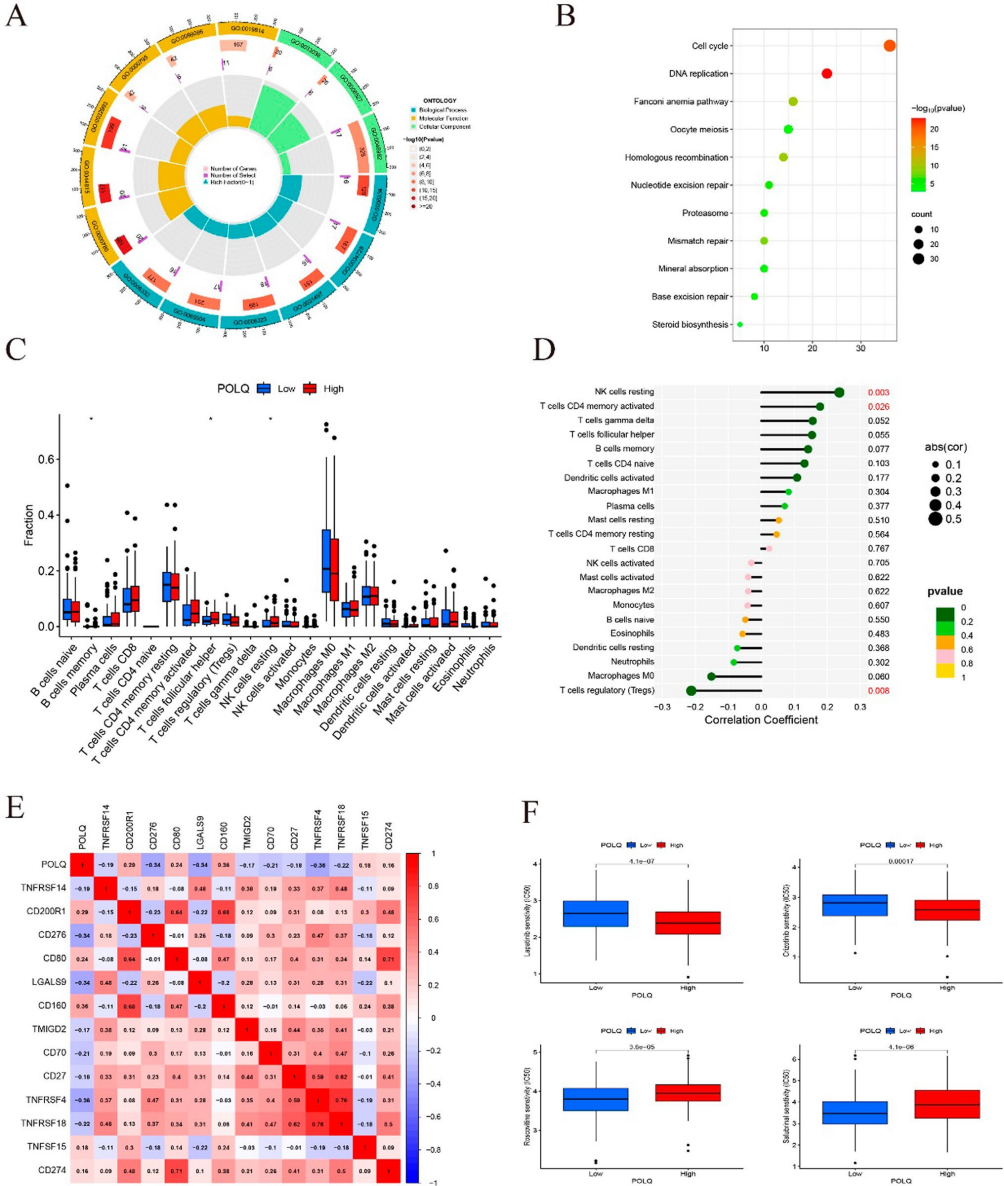
Exploring the association of POLQ with biological functions, immune infiltration and drug sensitivity in bioinformatics analysis. **A** The biological functions of POLQ determined by GO enrichment analysis. **B** POLQ-related signaling pathways analyzed by KEGG. **C**, **D** The association of POLQ with immune infiltration and immune checkpoints. **E**, **F** Relationship between POLQ and drug sensitivity. *P < 0.05, **P < 0.01, ***P < 0.001

To further investigate the potential biological behavior of POLQ and its co-expressed genes, GO enrichment analysis was performed to search for relevant biological pathways. The results showed that these genes were mainly enriched in protein heterodimerization activity, nucleosome organization, and protein‒DNA complexes (Fig. 2A). KEGG analysis showed that these genes were mainly enriched in the cell cycle, DNA replication, and Fanconi anemia signaling pathway (Fig. 2B).

In COAD, we explored whether POLQ was involved in the Tumor immune microenvironment (TIME) by analyzing immune correlations between the high and low POLQ expression groups. Human immune cell subpopulations were calculated for each COAD sample using the CIBERSORT algorithm. The results showed that the two groups had differences in memory B cells, follicular helper T cells, and resting NK cells (Fig. 2C, D). The correlation between POLQ and immune cells was analyzed and revealed that POLQ was positively correlated with CD200R1, CD80, CD160, TNFSF15, and CD274, which are immune checkpoints. In contrast, POLQ was negatively correlated with TNFRSF14, CD276, LGALS9, TMIGD2, CD70, CD27, TNFRSF4, and TNFRSF18 (Fig. 2E). In summary, POLQ is closely associated with immune checkpoints.

In the relationship between POLQ and cancer drugs, we found that the IC50 of lapatinib and crizotinib was lower in the high POLQ expression group, which suggested that the high POLQ expression group was more sensitive to the above drugs. However, the IC50 values of roscovitine and salubrinal in the high POLQ expression group were higher than those in the low POLQ expression group (Fig. 2F).

### The expression of POLQ is upregulated in CRC and associated with a poor prognosis for CRC patients

**Fig. 3:**
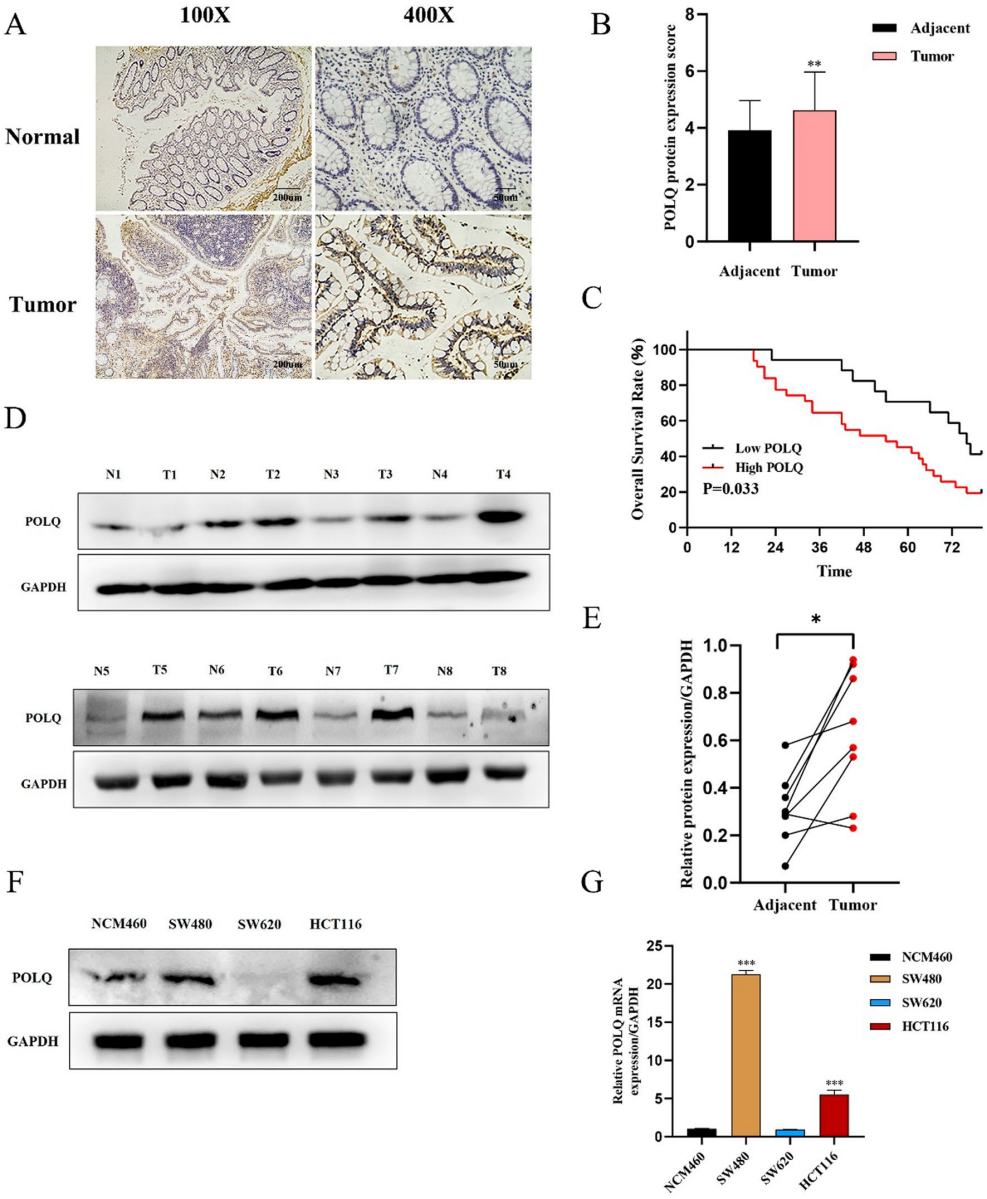
POLQ expression is upregulated in CRC cells and tissues. **A** The expression of POLQ in CRC tissues and para-carcinoma tissue was detected by immunofluorescenc (scale bars: 200 µm to 100 × , 50 µm to 400 × . **B** Qualification of POLQ staining in CRC tissues and para-carcinoma tissue. **C** Kaplan–Meier survival curves of 48 colorectal cancer patients with different POLQ expression levels were analyzed. **D** WB analysis results of POLQ in 8 pairs of CRC tissues and para-carcinoma tissue. **E** Paired *t* test of the western blot results. **F**, **G** Translational and transcriptional levels of POLQ in CRC cells lines (SW480, SW620, HCT116). *P < 0.05, **P < 0.01, ***P < 0.001

We evaluated the expression level of POLQ via IHC analysis on a microarray slide containing 48 pairs of CRC tissues and para-carcinoma tissue, which showed prominent upregulation in CRC tissues (P < 0.01; Fig. 3A, B). Eight pairs of fresh CRC tissues and para-carcinoma tissue were analyzed through western blotting, which presented similar outcomes with IHC (P = 0.01; Fig. 3D, E). Next, the mRNA transcription and protein translation levels of POLQ in NCM460 cell line and human CRC cell lines (SW480, SW620, HCT116) were analyzed by qRT‒PCR and WB analysis. Compared with NCM460 cells, the levels of mRNA transcription and protein translation were significantly higher in SW480 and HCT116 cells (P < 0.05), but no distinct difference was found in SW620 cells (Fig. 3F, G). Furthermore, the relationship between the level of POLQ expression and clinicopathological characteristics in 48 patients was analyzed. The level of POLQ expression was clearly different with age (P = 0.001) and gender (P = 0.017) but not with tumor size, histological type, differentiation, TNM stage, lymph node metastasis, depth of invasion, or venous invasion (Table 1). According to Kaplan‑Meier analysis, a high level of POLQ expression was positively associated with poor prognosis for CRC patients (P = 0.033; Fig. 3C). Univariate analysis showed that age and POLQ staining were independent risk factors for CRC prognosis. Additionally, the multivariate analysis showed that only age was an independent risk factor for OS (Table 2). All these findings suggest that POLQ is highly expressed in CRC tissues and cells and is markedly associated with poor prognosis.

**Table 1:** The correlation between POLQ expression and the clinical parameters in patients with CRC

Parameter	All case	POLQ expression		χ^2^	P value
	(n = 48)	Low(17)	High(31)		
Age (years)					
≤ 65	18	12	6	12.296	0.001
> 65	30	5	25		
Gender					
Female	20	11	9	5.749	0.017
Male	28	6	22		
Tumor size (cm)					
< 5	13	6	7	0.498	0.269
≥ 5	35	11	24		
Histological type differentiated					
Low	20	6	11	0.001	0.99
High	28	11	20		
TNM stage					
I/II	22	9	23	2.231	0.135
III/IV	16	8	8		
Lymph node metastasis					
Negative	22	9	23	2.231	0.135
Positive	16	8	8		
Depth of invasion					
T1/T2	8	2	6	0.698	0.405
T3/T4	40	15	25		
Venous invasion					
Negative	36	11	25	0.759	0.384
Positive	12	6	6

**Table 2:** Univariate and multivariate analysis of factors associated with OS

Factors	Univariate analysis		Multivariate analysis	
	HR (95% CI)	P-value	HR (95% CI)	P-value
Age (years)	0.277 (0.124–0.618)	0.002	0.328 (0.135–0.795)	0.014
(≤ 65 vs > 65)				
Gender	0.579 (0.277–1.208)	0.145		NA
(female vs male)				
Tumor size (cm)	1.204 (0.573–2.529)	0.642		NA
(< 5 vs ≥ 5)				
Histological type differentiated	0.589 (0.281–1.235)	0.161		NA
(low vs high)				
TNM stage	1.144 (0.557–2.348)	0.715		NA
(I/II vs III/IV)				
Lymph node metastasis	1.144 (0.557–2.348)	0.715		NA
(Negative vs positive)				
Depth of invasion	2.107 (0.739–6.004)	0.163		NA
(T1/T2 vs T3/T4)				
Venous invasion	0.427 (0.150–1.214)	0.11		NA
(negative vs positive)				
POLQ staining	0.418 (0.193–0.901)	0.026	0.693 (0.297–1.617)	0.396
(low vs high)				

*HR* hazard ratio, *95% CI* confidence interval, *NA* not adopted

### POLQ knockdown inhibits the proliferation, migration, and invasion of CRC cells

**Fig. 4:**
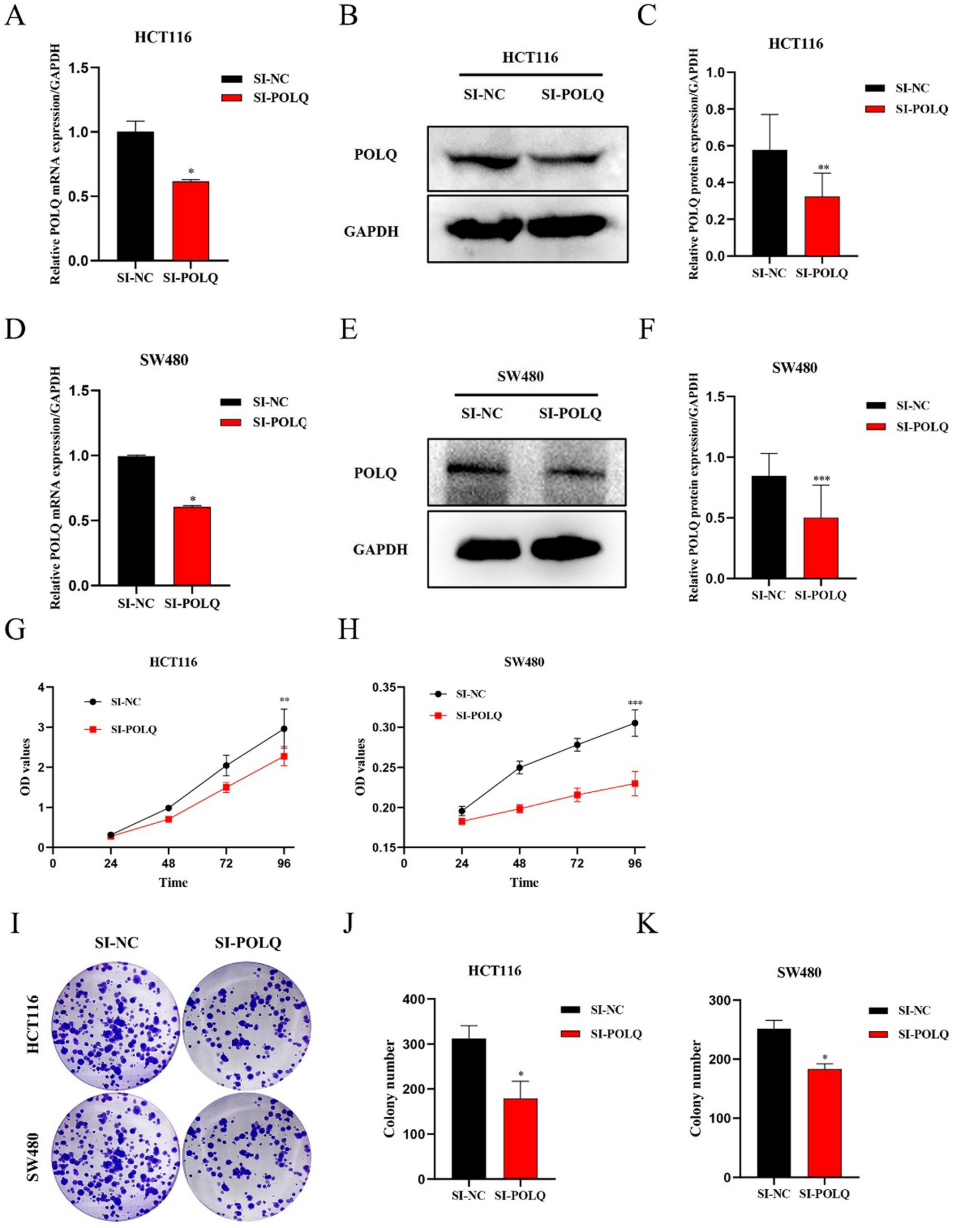
POLQ knockdown suppresses the proliferation ability of CRC cells The interference efficiency of siRNAs in HCT116 (**A**, **B**, **C**) and SW480 (**D**, **E**, **F**) cells was measured by qRT‒PCR and WB. CCK8 assay was used to detect the effect of POLQ knockdown on the proliferation of HCT116 (**G**) and SW480 (**H**) cells. **I**, **J**, **K** Detection of the proliferation of HCT116 and SW480 cells after POLQ knockdown by colony formation assays. *P < 0.05, **P < 0.01, ***P < 0.001

**Fig. 5:**
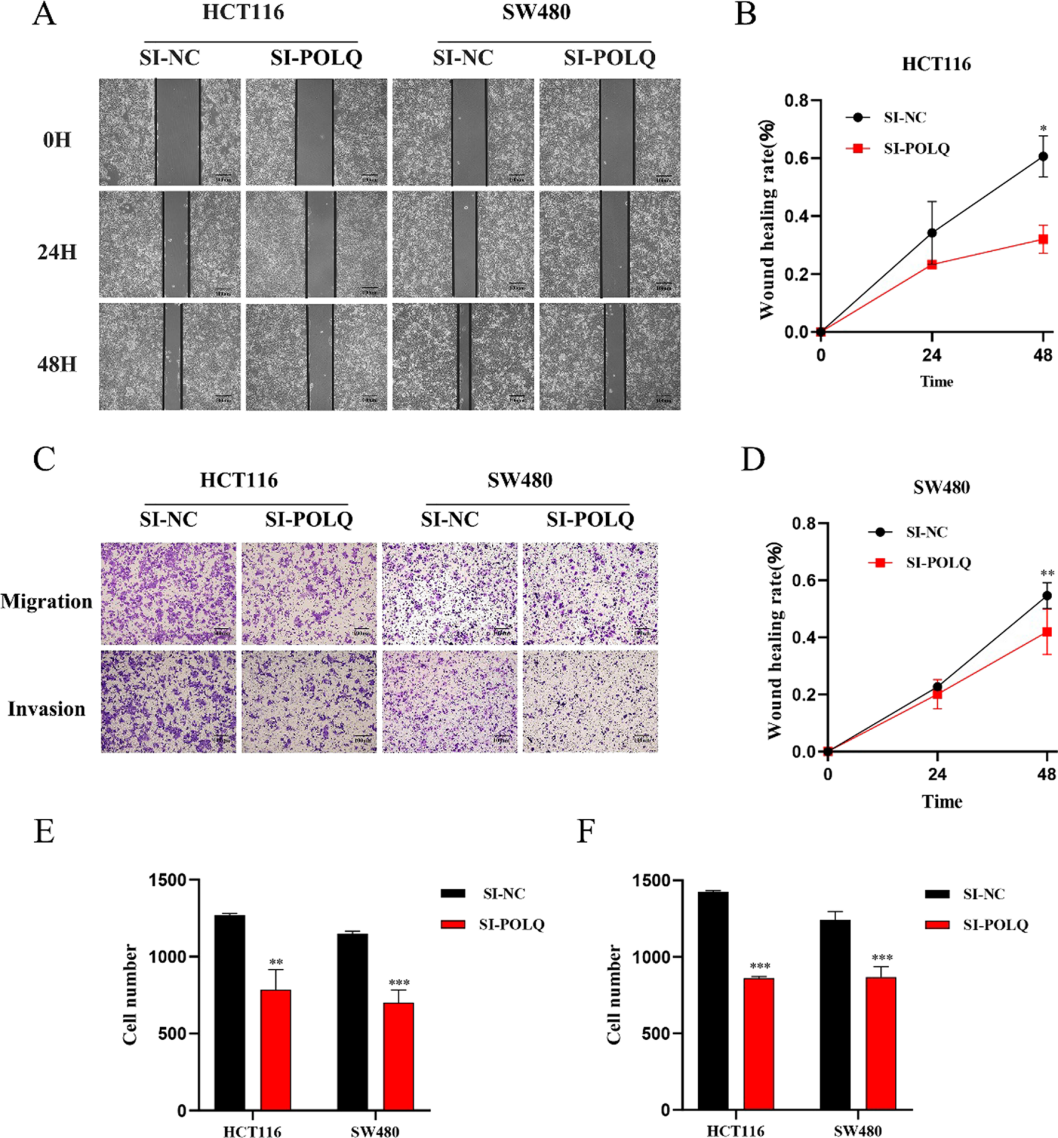
POLQ knockdown suppresses the migratory and invasive abilities of CRC cells (**A**, **B**, **D**) Scratch wound healing assay was used to investigate the cell migration ability of HCT116 and SW480 cells (scale bar: 100 µm). (**C**, **E**, **F**) Migratory and invasive abilities of HCT116 and SW480 cells were investigated by Boyden chamber assay (scale bar: 100 µm). *P < 0.05, **P < 0.01, ***P < 0.001

To obtain POLQ knockdown colorectal cancer cell models, siRNAs were separately transfected into SW480 and HCT116 cell lines. The results of qRT‒PCR and western blot analysis showed that the translational and transcriptional levels of POLQ were clearly decreased after siRNA transfection, which indicated that the POLQ knockdown cell model was successfully established (Fig. 4A–F). To analyze the effect of POLQ on the proliferation capacity of CRC cells, CCK-8 and colony formation assays were performed on SW480 and HCT116 cell lines, respectively. The results of CCK8 assay showed that the OD values were significantly lower in the POLQ knockdown group (Fig. 4G, H). Similarly, the results of colony formation assay showed that the proliferation function in the POLQ knockdown group was clearly downregulated (Fig. 4I-K). We performed scratch wound healing and Boyden chamber assays to verify the effect of POLQ knockdown on the migration and invasion abilities of CRC cell lines. In the scratch wound healing assay, two groups showed no significant difference in healing rate at 24 h. However, the POLQ knockdown group demonstrated a significantly lower healing rate at 48 h in both SW480 and HCT116 cells (Fig. 5A, B, D). Furthermore, the migration and invasion assays displayed a distinctly decreased number of cells passing through the Matrigel-coated membrane in the POLQ knockdown group (Fig. 5C, E, F). These results indicated that POLQ knockdown markedly inhibited the proliferation, migration, and invasion abilities of CRC cells.

### POLQ knockdown induced cell cycle arrest in CRC

**Fig. 6:**
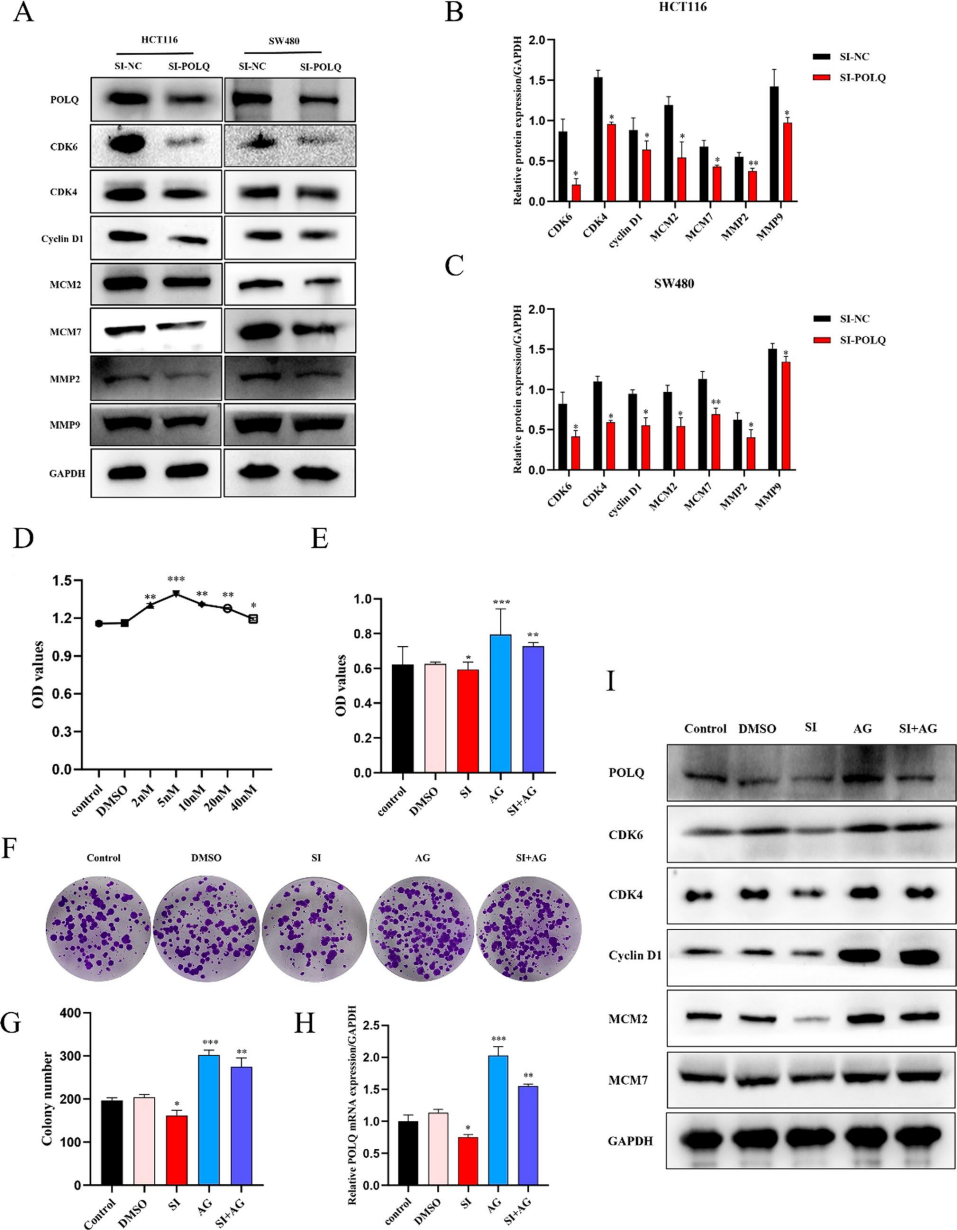
POLQ silencing affects the cell cycle signaling pathway in CRC (**A**, **B**, **C**) WB was used to detect the expression of CDK6, CDK4, cyclin D1, MCM2, MCM7, MMP2, and MMP9 in negative control (NC) cells and POLQ-knockdown HCT116 and SW480 cells. **D** CCK8 assay was used to assess the proliferation of HCT116 cells treated with various concentrations of agonist. **E** CCK8 assay was used to detect the effect of agonist treatment and POLQ knockdown on the proliferation of HCT116 cells. **F**, **G** Colony formation assay was used to analyze the proliferation ability of HCT116 cells after agonist treatment and POLQ knockdown. **H** The transcriptional level of POLQ in HCT116 cells treated with agonist and siRNAs was measured by qRT‒PCR. **I** The expression levels of POLQ, CDK6, CDK4, cyclin D1, MCM2 and MCM7 were assessed by WB after treatment with agonist and siRNAs in HCT116 cells. *P < 0.01, ***P < 0.001, ****P < 0.0001. (Control group: none; DMSO group: DMSO only; SI group: siRNAs only; AG group: agonist + DMSO; SI + AG group: siRNAs + agonist + DMSO)

The uncontrolled cell cycle is an essential feature of colorectal cancer [11]. To characterize the relationship between POLQ and the cell cycle signaling pathway in CRC, we analyzed the expression levels of various essential genes in regulating the cell cycle signaling pathway after POLQ knockdown in CRC cells. After POLQ knockdown, genes including CDK6, CDK4, and cyclin D1 that possess a potential role in regulating the G1/M phase were distinctly downregulated via WB analysis. Additionally, the expression levels of MCM2 and MCM7 belonging to the S/M phase were also significantly decreased after POLQ knockdown. Furthermore, POLQ knockdown distinctly reduced the expression levels of MMP2 and MMP9, which were associated with tumor metastasis and invasion. (Fig. 6A–C).

DMT-dC (ac) phosphoramidite is a modified phosphoramidite monomer that can be used to synthesize oligonucleotides and phosphoramidites and is an activator of the cell cycle signaling pathway. Therefore, we used it to further explore the relationship between POLQ and the cell cycle signaling pathway in promoting the progression of colorectal cancer. The results showed that MT-DC (ac) phosphoramidite reached the optimal concentration of 5 nM in regulating cell proliferation via CCK8 analysis (Fig. 6D). CCK8 and colony formation assays also confirmed that MT-DC (ac) phosphoramidite can significantly promote the proliferative function of CRC cells and had the maximal effect at a concentration of 5 nM. Moreover, the cell proliferation capacity in the SI + AG group was significantly higher than that in the control group but still lower than that in the AG group (Fig. 6E, G, H). The expression and transcription levels of POLQ in the AG group were significantly upregulated compared with those in the control group. Similarly, the expression and transcription levels of POLQ in the SI + AG group were considerably upregulated compared with those in the control group but were still lower than those in the AG group (Fig. 6F, I). Interestingly, the expression levels of CDK6, CDK4, cyclin D1, MCM2, and MCM7 in the AG group were significantly increased with the influence of the agonist. The expression levels of the above proteins in the SI + AG group were also clearly upregulated but were still lower than those in the AG group (Fig. 6F). Collectively, these results suggest that POLQ could affect colorectal cell function and progression by affecting the cell cycle signaling pathway.

## Discussion

POLQ is a DNA polymerase associated with error-prone translesion DNA synthesis and error-prone repair of DNA double-strand breaks (DSBs) [12]. Meanwhile, POLQ acted as a regulator of chromosomal break repair and replication stress response under the intervention of RAD5 [13]. Masuda [14] revealed that higher relative POLQ expression was found in various cancers including stomach, lung, and colon cancer, and discovered poor prognosis in patients with POLQ overexpression. Moreover, Qi [6] indicated that POLQ knockdown interferes with the development and progression of hepatocellular carcinoma by regulating cell proliferation, apoptosis, and migration. Overexpression of POLQ increased somatic mutation load and Polo-like kinase 4 (PLK4) overexpression, which induces centrosome amplification and is associated with an advanced pathologic stage in lung adenocarcinoma [7]. Lemée [8] found that POLQ was significantly up-regulated in breast cancer tissues, and the up-regulation of POLQ expression level was associated with poor prognosis of patients (the risk of death in patients with high POLQ expression was 4.3 times higher than that in patients with low expression). Regrettably, few studies have investigated the function of POLQ in colorectal cancer and its regulation of tumor progression.

Presently, our study proved that POLQ was overexpressed in CRC tissues and cells and served as an independent risk factor via the analysis of clinical data from 48 CRC patients. Meanwhile, the high expression level of POLQ was associated with male and older than 65 years of age in CRC patients. Univariate analysis showed that high POLQ expression and age over 65 were independent risk factors for CRC patients. Next, we analyzed the effect of POLQ knockdown on CRC cells in vitro. Results showed that POLQ knockdown markedly weakened the proliferation, metastasis, and invasion of CRC cells. Moreover, we found a relationship between the cell cycle signaling pathway and POLQ through bioinformatics analysis.

The cell cycle is a series of events that drive a cell to divide into two new daughter cells. The typical cell cycle in eukaryotes consists of four phases, including G1, S, G2, and M phase [15]. Interestingly, the progression of cell cycle is regulated by CDKs and their cyclin subunits. CDK4 and CDK6 in the CDK family and their involved D-type cyclins are key components of the cell cycle machinery by phosphorylating and inactivating retinoblastoma proteins to drive the G1 to S phase transition [16, 17]. Simultaneously, previous studies have demonstrated that the cyclin D-CDK axis plays a crucial role in cancer by controlling cell proliferation, senescence, migration, apoptosis, and angiogenesis and revealed that the abnormal regulation of cyclin Ds-CDK axis is a hallmark of tumors [16, 18, 19]. Furthermore, Three CDK4/6-specific inhibitors, palbociclib (Pfizer), ribociclib (Novartis), and abemaciclib (Eli Lilly), have been approved by the United States Food and Drug Administration (FDA) and used in combination with other treatment regimens for various cancers [20, 21]. Interestingly, our results showed that the expression levels of CDK4, CDK6, and cyclin D1 in CRC cells were visually reduced after POLQ knockdown, which suggested that POLQ knockdown affects the cell cycle process and may be a potential factor in cancer therapy. Initially, MCMs were identified as genes that played critical roles in maintaining extrachromosomal DNA in Saccharomyces cerevisiae [22]. MCM2-MCM7, as members of the MCM family, form a heterohexameric complex that acts as a replicative DNA helicase to unwind the DNA duplex template during DNA replication in eukaryotic cells [23, 24]. MCMs were deemed to be markers of cell proliferation in the cell cycle. Increased levels of MCMs indicate the proliferation of malignant cells. Moreover, much evidence has suggested that MCMs play a role in predicting tumor progression and prognosis [25]. MCM2 and MCM7, defined as CRC-related genes, were overexpressed and associated with proliferative capacity, tumor histological grade, lymph node metastasis and vascular invasion in CRC [26]. Liu [27] indicated that MCM2 knockdown led to an arrest in the cell cycle, along with suppression of cell growth but an acceleration of cell apoptosis in HCT116 cell line. Similarly, our results revealed that POLQ knockdown reduced the expression levels of MCM2 and MCM7, which may contribute to the inhibition of DNA replication and thus impair the progression of S phase.

Matrix metalloproteinases (MMPs) are a group of zinc endopeptidases that play a critical role in the decomposition of extracellular matrix (ECM) components and basement membrane (BM) [28]. The expression of MMPs is uncontrolled in most types of cancers and is closely associated with tumor progression, including proliferation, invasion, epithelial–mesenchymal transition (EMT), metastasis, and angiogenesis [29]. MMP2 and MMP9, members of the MMP family, are often involved in poor prognosis and linked to tumor invasion and metastasis in CRC y[30]. Thus far, our results revealed that POLQ knockdown distinctly suppresses the expression of MMP2 and MMP9, which could indicate that the progression of CRC cells was indeed attenuated.

Significant clinical differences were observed between high and low expression of POLQ in patients, which may make POLQ a good prognostic marker and a potential clinical therapeutic target. Currently, multiple institutions have developed inhibitors for POLQ. The use of poly ADP-ribose polymerase 1 (PARP1) has been very common in the treatment of breast cancer and ovarian cancer, and its role may be to prevent the repair of fatal single strand breaks and double strand breaks in some genetic backgrounds. The current research results indicate that targeting PARP1 combined with inhibiting POLQ may be an attractive therapeutic option for HR deficient tumors. Mouse embryonic fibroblasts lacking both BRCA1 and POLQ exhibit increased chromosomal aberrations and radial chromosomal formation. Mouse embryonic fibroblasts lacking both BRCA1 and Pol θ exhibit increased chromosomal aberrations and radial chromosomal formation. These results indicate that HR deficient tumors rely on the repair of DSBs by POLQ, and can be effectively targeted for treatment by simultaneously knocking down POLQ and chemotherapy.

## Conclusion

We found that age and stage were closely related to the prognosis of CRC through biological information analysis, and the analysis of collected clinical data also showed that age was an independent risk factor for OS. There are a series of genes positively correlated with POLQ expression, such as KNTC1, ATAD5, XRCC2, GEN1, CEP152 and BRCA1. The transcription and expression of POLQ are significantly up-regulated in colorectal cancer tissues and cells. Furthermore, POLQ knockdown effectively inhibits the proliferation, metastasis, and invasion of CRC cells. POLQ could affect cell cycle progression by affecting some key genes in the cell cycle pathway including CDK6, CDK4, cyclin D1, MCM2 and MCM7. All results gained in this study may provide some theoretical basis for diagnosing and treating CRC.

## Data Availability

The data used to support the findings of this study are available from the corresponding authors upon request.
